# Does Intraperitoneal Chemotherapy Increase the Incidence of Anastomotic Leakage after Colorectal Cancer Surgery: A Systematic Review and Meta-Analysis

**DOI:** 10.1155/2021/9204373

**Published:** 2021-01-25

**Authors:** Yu Yang, Yuxuan Li, Xiaohui Du

**Affiliations:** Department of General Surgery, Chinese PLA General Hospital, Beijing 100853, China

## Abstract

**Purpose:**

To identify and evaluate the influence of intraperitoneal chemotherapy without hyperthermia (IC_wh_) to the incidence of anastomotic leakage (AL) after colorectal cancer surgery.

**Methods:**

A systematic review and meta-analysis were performed according to the Preferred Reporting Items for Systematic Reviews and Meta-Analyses in order to review all studies investigating the relationship between IC_wh_ and AL in patients undergoing colorectal surgery. The primary outcome was overall incidence rate of anastomotic leakage.

**Results:**

Four studies were included in the final review. IC_wh_ was associated with an overall increased risk of anastomotic leakage [OR 2.05 (1.06, 3.98), *P* = 0.03]. But there was no significant increased incidence rate when fluorouracil was implanted into the abdominal cavity for IC_wh_ [OR 2.48 (0.55, 11.10), *P* = 0.24].

**Conclusions:**

This meta-analysis provides some evidence to suggest IC_wh_ may increase the incidence of postoperative AL in colorectal cancer. However, fluorouracil implantation for IC_wh_ does not increase the risk of AL, which seems to be a relatively safe method of IC_wh_.

## 1. Introduction

Colorectal cancer ranks third in terms of incidence and second in terms of mortality among all cancers [1]. Appropriate patient selection with respect to surgical treatment options and the use of multimodality therapy could substantially affect recurrence and survival [2]. Chemotherapy plays an important role in preventing cancer recurrence and metastasis. The conventional venous chemotherapy has poor targeting and systemic side effects are inevitable, so intraperitoneal chemotherapy (IC) has been paid more and more attention in recent years and has become a new treatment. Hyperthermic intraperitoneal chemotherapy (HIPEC) has been widely used, but based on advantages of straightforward operation and good tolerance, intraperitoneal chemotherapy without hyperthermia (IC_wh_) has become one of the important means of comprehensive treatment of colorectal cancers under the concept of enhanced recovery after surgery (ERAS). Regarding the relationship between IC_wh_ and postoperative complications, especially the occurrence of anastomotic leakage (AL), the results of different studies are not consistent [3, 4]. Therefore, the main purpose of this meta-analysis is to provide better evidence for whether IC_wh_ increases the incidence of anastomotic leakage after colorectal cancer surgery.

## 2. Methods

### 2.1. Search Strategy

The following systematic review and meta-analysis were completed using the Preferred Reporting Items for Systematic Reviews and Meta-Analyses (PRISMA) guidelines [5]. Two investigators independently performed an electronic literature search (Yu Yang and Yuxuan Li) of PubMed, Embase, and Cochrane Library, which only included research published from inception to June 1, 2020. We used the combined terms as follows: either MeSH or title/abstract relating to (“intraperitoneal chemotherapy”), (“leakage” or “leak” or “fistula” or “perforation” or “break”), and (“colorectal” or “colonic” or “rectal” or “sigmoid”), restricting to English. In addition, the investigators performed a manual search of the reference lists of all potential articles' full texts.

### 2.2. Selection Criteria and Exclusion Criteria

The following criteria needed to be met for the study to be included. (1) Patients suffering from colonic or rectal cancer were treated with a colonic or rectal resection and anastomosis. (2) Administration of intraperitoneal chemotherapy was compared to a control group without intraperitoneal chemotherapy administration. (3) The evaluation indicators of the study included the occurrence of postoperative anastomotic leakage. (4) The study type is randomized controlled trials, cohort studies, and prospective observational studies. (5) Only studies published in the English language were included. The exclusion criteria included the following. (1) The mode of intraperitoneal chemotherapy is hyperthermic intraperitoneal chemotherapy (HIPEC). (2) Data were published in multiple studies from the same institution. (3) Case reports, case controlled studies, reviews, letters, and articles lacking necessary data for calculation were excluded. (4) The object of the study is isolated human cells, isolated human organs, and animals.

### 2.3. Risk of Bias Assessment

The methodological quality assessment of the RCTs will be evaluated using the Cochrane collaboration's risk of bias tool [6]. The methodological index for nonrandomized studies (MINORS) will be used to evaluate the methodological quality of the included nonrandomized comparative studies [7]. The studies should be moderate or high according to the Cochrane risk of bias tool or MINORS criteria.

### 2.4. Data Extraction

Data extraction was completed independently by two investigators (Yu Yang and Yuxuan Li). Any disagreements will be resolved through discussion. We selected the most recent one for multiple publications from the same study. A standard data collection form was used with the following information: the first author, year of publication, country, study design, number of participants that underwent intraperitoneal chemotherapy and anastomotic leakage number, number of participants without intraperitoneal chemotherapy and anastomotic leakage number, mean age, gender, chemotherapeutic drugs, localization of tumor, TNM stage, and tumor differentiation grade.

### 2.5. Statistical Analysis

Statistical analyses will be performed using RevMan version 5.3 (The Cochrane Collaboration, Copenhagen, Denmark). The calculated pooled risk ratios (ORs) are used to compare incidence of AL between nonthermal intraperitoneal chemotherapy users versus nonusers between subgroups.*P* < 0.05 was considered significant for all of the analyses. Interstudy heterogeneity was assessed using the *I*^2^ statistic. The random effects model will be applied if there is significant statistical heterogeneity; otherwise, the fixed effects model will be applied. Statistical heterogeneity is considered significant with an *I*^2^ test of 50% or higher.

## 3. Results

### 3.1. Study Selection and Characteristics

**Figure 1:**
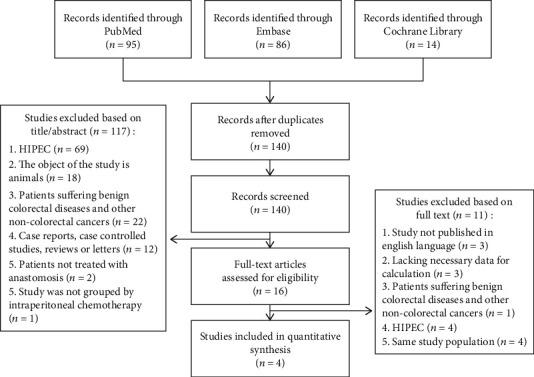
Flow diagram of study selection.

A total of 4 studies involving 4 trials were identified for inclusion in the review [3, 4, 8, 9]. The search of PubMed, Embase, and Cochrane Library databases provided a total of 195 citations. After adjusting for duplicates, 140 remained. Of these, 117 studies were discarded after reviewing the titles and abstracts by selection and exclusion criteria. Eleven additional studies were discarded because the full text of the study did not meet the selection requirement. The full text of the remaining 4 citations was examined in more detail and data included in the systematic review and meta-analysis (Figure 1).

Finally, 1499 participants were involved. All studies were published in English with three originating from East Asia and one from America; two were RCT design and the other two were retrospective cohort design (Table 1).

### 3.2. Risk of Bias within Studies

The quality assessment is shown in Table 1; in none of the studies were the investigators blinded, none of the studies did sample size estimation, one retrospective cohort study was deemed moderate risk because it did not even provide baseline statistics, and other three studies were graded high risk according to the Cochrane risk of bias tool or MINORS criteria.

### 3.3. Results of Individual Studies

**Figure 2:**
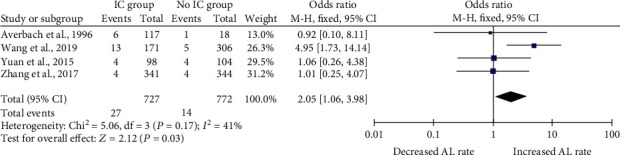
IC_wh_ post colorectal surgery and anastomotic leak rate.

IC_wh_ was associated with a significantly higher anastomotic leak rate in one of the four studies. Synthesized using the Mantel-Haenszel (M-H) analysis, OR with fixed effects model, the overall anastomotic leak rate was found to be significantly higher in the IC group [OR 2.05 (1.06, 3.98), *P* = 0.03]. Heterogeneity as defined by *I*^2^ statistics was lower than 50% (41%) (Figure 2).

**Figure 3:**
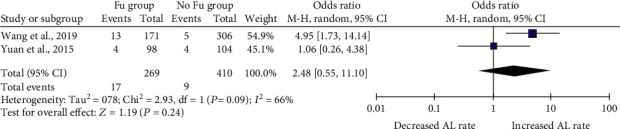
Fu implantation for IC_wh_ post colorectal surgery and anastomotic leak rate.

In Wang et al.'s study [4], totally 171 patients received IC_wh_, mainly 157 were treated with fluorouracil implants alone, and another 14 were treated with lobaplatin or both. According to the same period and same institution about IC_wh_ with lobaplatin, data presented that only 2% (1/50) had anastomotic leakage; we regard this set of data as the use of fluorouracil only and then combine with the data in Yuan et al.'s [3] study to analyze the relationship between the use of fluorouracil implanted into the abdominal cavity as IC_wh_ and the occurrence of AL after colorectal cancer surgery. Synthesized using M-H analysis, OR with random effects model, the overall anastomotic leak rate was not found to be higher in the IC group [OR 2.48 (0.55, 11.10), *P* = 0.24]. Heterogeneity as defined by *I*^2^ statistics was higher than 50% (66%) (Figure 3).

### 3.4. Risk of Bias across Studies

**Figure 4:**
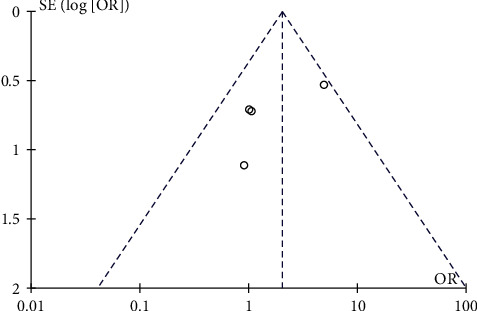
Funnel plot (IC_wh_ vs. no IC_wh_).

Compared with the control group, lower heterogeneity was found in the overall IC_wh_ analysis (*I*^2^ = 41%). This heterogeneity was further assessed using a funnel plot (Figure 4). Considering the low number of studies included in our analysis, the overall shape of our funnel plot is still fairly symmetrical.

## 4. Discussion

This is the first meta-analysis describing the effect of IC_wh_ on the incidence rate of anastomotic leakage in colorectal cancer surgery. Overall, the evidence in this study indicates that IC_wh_ increases the incidence of anastomotic leakage. However, further analysis of the implantation of fluorouracil in the abdominal cavity for intraperitoneal chemotherapy did not increase the risk of postoperative anastomotic leakage.

Postoperative recurrence is an important factor affecting the prognosis of colorectal cancer patients, and chemotherapy is an important means to prevent postoperative cancer recurrence and metastasis [10]. Within 7 days after primary lesion resection, it is the best time to kill residual cancer cells and micrometastases, because in this period the residual cancer cells are most sensitive to intraperitoneal chemotherapy [11]. Venous chemotherapy needs to be carried out after the postoperative recovery period, and the guidelines recommend that it be carried out 4 weeks after surgery [12, 13], often missing the most sensitive stage. Precisely, IC can inhibit and kill tumor cells in this sensitive period. Some studies have shown that IC can improve the prognosis of colorectal patients.

However, the adverse effects of chemotherapy on postoperative anastomotic healing cannot be ignored, the role of chemotherapeutic agents is to inhibit the proliferation of cells [14], and the process of wound healing is the most active period of cell proliferation; animal experiments have also shown that chemotherapeutic agents inhibit wound healing [15, 16]. The results of previous clinical studies on whether IC increases the incidence of anastomotic leakage are not consistent [3, 4]. In this study, the synthesized results indicate that IC_wh_ increases the incidence of AL after colorectal cancer surgery. It is suggested that we should alert the occurrence of anastomotic leakage after intraperitoneal chemotherapy.

Adjuvant chemotherapy has an established role for patients with high-risk stage II and stage III colon cancer, reducing the risk of recurrence and death by approximately 20–30% with fluoropyrimidine monotherapy [17]. One study only used fluorouracil implanted in the abdominal cavity as IC_wh_ suggested this method did not increase the incidence of AL and other related complications and at the same time improved the prognosis of patients [3]. Our synthesized analysis result also suggested that fluorouracil implantation does not increase the risk of AL. Therefore, we infer that this may be a relatively safe and effective method of IC_wh_.

This study has some inevitable limitations. The major one is due to the fact that we focus on the limited direction of IC_wh_ and AL in colorectal cancers, there are only 4 studies included, with 2 retrospective designs included. Only one study suggested that IC_wh_ significantly increased the incidence of AL. Secondly, due to the limited sample size of the patients in the four studies, we did not synthesize the baseline information of patients with different cancer stages, perioperative neoadjuvant therapy, tumor location, surgical methods, and so on; also, we did not analyze these cancer risk factors by subgroup. Furthermore, because the methods of IC_wh_ in the four studies are not unified and the data on the type, usage, and dosage of chemotherapeutic agents are incomplete, it is impossible to analyze the dose-effect relationship of chemotherapeutic agents. In general, like all studies that rely on observed data, due to a variety of confounding factors and biases, our study cannot get a clear causal relationship; there is still a need for more prospective, well-designed, and large-sample studies to provide more reliable evidence.

## 5. Conclusions

This meta-analysis provides some evidence to suggest that IC_wh_ may increase the incidence of postoperative AL in colorectal cancer. However, fluorouracil implantation for IC_wh_ does not increase the risk of AL, which seems to be a relatively safe method of IC_wh_. We suggest that surgeons should pay attention to the related complications of IC_wh_, especially anastomotic leakage.

## Figures and Tables

**Table 1:** Basic characteristics of the included studies.

Study	Type of study	Years of the study	IC method	Patient number		AL number		Risk of bias
				IC	No IC	IC	No IC	
Zhang et al., 2017	RCT	2011.01–2016.01	300 mg/m^2^ 5-FU poured into the abdominal cavity	341	344	4 (1.2%)	4 (1.2%)	High
Yuan et al., 2015	RCT	2007.06–2008.07	Implant 5-Fu (600 mg) into the abdominal cavity	98	104	4 (4.1%)	4 (3.8%)	High
Wang et al., 2019	Retrospective cohort	2016.09–2017.09	Implant Fu (500-1000 mg) into the abdominal cavity or poured lobaplatin (60 mg) into the abdominal cavity	171	306	13 (7.6%)	5 (1.6%)	High
Averbach et al., 1996	Retrospective cohort	1988–1994	Mitomycin C (10 mg/m^2^ BSA) and 5-Fu (15 mg/kg body weight) poured into the abdominal cavity	117	18	6 (5%)	1 (6%)	Moderate

RCT: randomized controlled trial; IC: intraperitoneal chemotherapy; Fu: fluorouracil; BSA: body surface area.
